# Treatment Outcomes of Multiple Myeloma in Developing Countries: A Systematic Review and Meta-Analysis

**DOI:** 10.46989/001c.144582

**Published:** 2025-10-08

**Authors:** Jehad Almasri, Bashar Hasan, Zin Tarakji, Faris W. Naffa’, Lynn Warner, Hira Mian, Rajshekhar Chakraborty, Ghulam Rehman Mohyuddin, Zaid Abdel-Rahman, Samer Al Hadidi

**Affiliations:** 1 University of Cincinnati Cancer Center, Cincinnati, OH, USA; 2 Evidence-Based Practice Center, Mayo Clinic, Rochester, MN, USA; 3 Mayo Clinic, Rochester, MN, USA; 4 Internal Medicine Department, Ascension Saint Agnes, Baltimore, MD, USA; 5 Health Science Library, University of Cincinnati, Cincinnati, OH, USA; 6 McMaster University, Hamilton, Ontario, Canada; 7 Herbert Irving Comprehensive Cancer Center, Columbia University Irving Medical Center, New York, NY, USA; 8 Huntsman Cancer Institute, University of Utah, Salt Lake City, UT, USA; 9 King Hussein cancer center, Amman, Jordan; 10 Myeloma Center, Winthrop P. Rockefeller Cancer Institute, University of Arkansas for Medical Sciences, Little Rock, AR, USA

**Keywords:** Multiple myeloma, developing countries, global, Hematopoietic Stem Cell Transplantation.

## Abstract

Multiple myeloma (MM) treatment outcomes in developing countries may be impacted by resource constraints. This systematic review and meta-analysis evaluated efficacy outcomes of MM treatments across developing regions. Comprehensive searches in five major databases identified 37 eligible studies from Asia, Africa, Latin America, and Eastern Europe. The Newcastle-Ottawa Scale was used to assess risk of bias. Newer novel agents including daratumumab, carfilzomib, and pomalidomide showed limited use across studies. For patients receiving autologous stem cell transplantation (ASCT), the pooled overall survival rate at longest follow-up (2.5-12.5 years) was 62% (95% CI: 48-75%), with high heterogeneity (I²=92%), while the progression-free survival rate at longest follow-up (3-8 years) was 44% (95% CI: 23-67%). Comparative analyses demonstrated ASCT was associated with significantly superior 5-year survival compared to conventional chemotherapy (RR: 1.59; 95% CI: 1.38-1.82). Bortezomib-based regimens showed better outcomes than thalidomide-based therapies (HR for overall survival (OS) at 4 years: 0.73; 95% CI: 0.53-1.0) and alkylating agent-based regimens (HR: 0.48; 95% CI: 0.28-0.83). Despite resource limitations, ASCT and certain novel agents are associated with improved survival outcomes for MM patients in developing countries. However, substantial heterogeneity in outcomes suggests variability in healthcare infrastructure, treatment accessibility, and clinical expertise across these regions.

## INTRODUCTION

Multiple myeloma (MM), a complex hematologic malignancy characterized by the proliferation of plasma cells in the bone marrow, poses significant challenges to healthcare systems worldwide. While developed countries have seen considerable advancements in the diagnosis, treatment, and management of this disease, leading to improved survival rates and quality of life for patients, developing countries face a different reality. According to the World Health Organization (WHO), approximately 70% of all cancer-related deaths occur in low- and middle-income countries (LMICs), highlighting the disproportionate impact of cancer in these regions. Furthermore, a study revealed that while MM incidence rates are lower in developing countries compared to developed ones, the mortality-to-incidence ratio is substantially higher, indicating poorer outcomes and limited access to effective treatments.^1^

While individual studies from single centers or countries have reported outcomes, no prior analysis has synthesized this evidence to provide regional and global perspectives on MM care in resource-limited settings.

Access to diagnostic facilities is particularly challenging, with only 25% of LMICs having pathology services available, as reported by the International Agency for Research on Cancer (IARC).^2^ Additionally, the availability of treatments is severely restricted in many developing countries, with essential medications like proteasome inhibitors and immunomodulatory drugs often inaccessible, due to high costs or lack of procurement infrastructure.^3^ These disparities contribute to delayed diagnoses, advanced disease presentations, and suboptimal outcomes for MM patients in these regions.

The importance of studying MM outcomes in these regions cannot be overstated. As the global burden of cancer shifts, with an increasing proportion of cases occurring in LMICs, understanding the specific challenges and outcomes related to MM in such settings becomes crucial. This systematic review seeks to illuminate the outcomes of MM in developing nations, where limited access to healthcare resources, economic constraints, and a lack of awareness significantly impact disease prognosis.

## METHODS

### Search Strategy

A comprehensive search strategy was developed and performed by a librarian (LW). Databases used for the search were Embase via Elsevier, PubMed, Scopus, CINAHL, and CENTRAL. All searches were run from inception to March 13, 2023. Key terms in the search included “multiple myeloma”, “plasma cell myeloma”, “myelomatosis”, “developing countries”, “developing nation”, “underdeveloped country”, “less developed country”, “third world countries”, “low-income countries”, “middle income countries”, “LMICs”, “least developed countries”, “lower-middle-income countries”, and “developing area”. Records were uploaded to and deduplicated through Rayyan (https://rayyan.ai/). A total of 448 records were identified, and after duplicates were removed, 244 articles remained for screening.

### Selection Criteria

Prospective and retrospective studies were included if they: (1) were conducted in countries classified as developing by the World Bank at the time of study; (2) reported efficacy treatment outcomes for patients with MM. We included systematic reviews to cross the references and ensure inclusion of all eligible studies. We excluded case reports, reviews, and editorials. Studies were excluded if they represented developed-country treatment protocols with full access to novel agents, even if conducted in developing countries, as they would not reflect typical resource constraints faced in these settings.

### Data Screening and Extraction

Two independent reviewers screened abstracts/full texts (JA, FN) in a duplicate manner. Data extraction was performed by four reviewers (BH, ZT, FN, JA) using excel sheet with planned extracted data . Extracted information included: study characteristics (author, publication year, country, study design); patient demographics (age, gender, disease stage, genetic abnormalities); treatment details (regimen, dosage, duration); and outcomes (OS, PFS, response rates). Discrepancies were resolved through discussion with a third reviewer (SA).

### Risk of Bias Assessment

Risk of bias was assessed using an adaptation of the Newcastle-Ottawa Scale for observational studies, evaluating selection, comparability, and outcome domains. Studies were categorized as having low, moderate, or high risk of bias based on their scores. The majority of included studies (27/37) were assessed as having low risk of bias, particularly in the domains of representativeness of the cohort, ascertainment of exposure, and outcome assessment. Ten studies had unclear or high risk of bias in at least one domain, primarily related to adequacy of follow-up or insufficient detail to allow replication.

### Statistical Analysis

We anticipated significant heterogeneity given the diverse healthcare systems, economic conditions, and treatment accessibility across developing countries. This heterogeneity was considered clinically meaningful and reflective of real-world conditions rather than a limitation, as it provides insights into the range of outcomes achievable under different resource constraints.

For comparative studies, we calculated the risk ratio (RR) for binary outcomes such as OS and PFS, estimating 95% confidence intervals (CIs) and pooling across studies using the DerSimonian and Laird random effects model. For noncomparative studies, we calculated overall event rates and transformed them using the Freeman-Tukey double arcsine method, then pooled using the DerSimonian and Laird random effects model. The random effects model was chosen a priori due to anticipated heterogeneity across study populations and settings in different countries and institutions. Heterogeneity was evaluated using the I² index and Cochran’s Q test. Forest plots were generated to visualize results, displaying individual study effects, pooled estimates, and 95% CIs. All statistical analyses were conducted using R version 4.4.0 (The R Foundation for Statistical Computing, Vienna, Austria).

## RESULTS

### Study Selection and Characteristics

**Figure 1.**
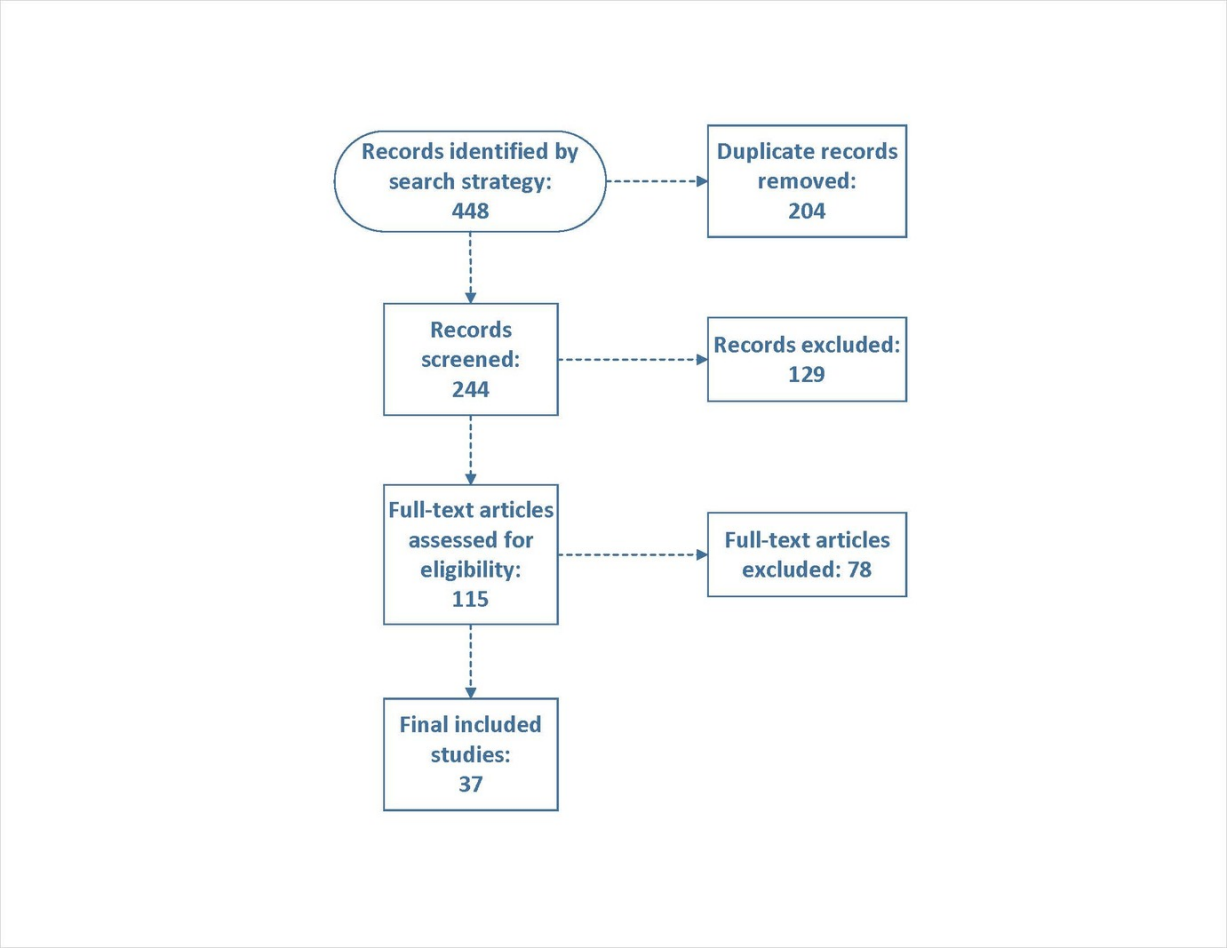
PRISMA flow diagram of study selection process.

Our initial search identified 448 records, of which 204 were removed as duplicates. After screening 244 titles and abstracts, 115 full-text articles were assessed for eligibility. Ultimately, 37 studies (2 prospective and 35 retrospective studies) met our inclusion criteria and were included in the meta-analysis **(Figure 1).**

The 37 included studies were conducted across 16 developing countries, with the most common being India (n=10), Brazil (n=4), Mexico (n=4), Algeria (n=3), Colombia (n=3), and Sri Lanka (n=3), along with studies from other countries across Asia, Africa, and Latin America. The studies collectively included 4,818 patients with MM, with individual sample sizes ranging from 10 to 816 patients. The median age of patients ranged from 48 to 66 years, with male predominance in most studies (55-70%). Disease staging varied, with a considerable proportion of patients presenting with advanced disease (ISS stage III: 28-58%). The most common myeloma subtype was IgG (50-72%), followed by IgA (10-25%) and light chain disease (8-18%). **Table 1** summarizes the characteristics of included studies.

**Table 1.** Characteristics of the included studies.

Author, year	Country where study was conducted	Inclusion Criteria	Exclusion Criteria	Length of follow-up	Intervention name	Describe Intervention
Hewamana, 2022	Sri Lanka	Patients who received PBSCT at the center	Patients with plasmablastic lymphoma, plasma cell leukemia, and creatinine clearance less than 30ml/min at presentation were not included in the final analysis	38.33 months	Peripheral blood stem cell transplant (PBSCT)	Melphalan 100mg/m2 was used for conditioning prior to PBSCT. HSC dose (per Kg): Median (IQR, Range): 4.25 (4.21, (1.64, 17.22))
Reddy, 2022	India	Patients with relapsed MM post ASCT who were treated with a salvage regimen	Patients with incomplete treatment details (6 out of 51 patients were excluded)	Median follow-up of 8.1 months (Interquartile Range, IQR: 3.1–26.7 months)	Salvage regimens post ASCT relapse	Salvage regimens used in treatment post ASCT relapse, specifically mentioning Pomalidomide-based salvage therapy (VPd, PCd) for 18 (39.2%) patients and VRd for 14 (30.5%) patients
Sasmith, 2022	India	Newly diagnosed MM patients	NR	5 years	Various chemotherapy regimens	Various chemotherapy regimens and ASCT, specifically: 1) Cyclophosphamide, dexamethasone, and thalidomide (34%). 2) Bortezomib, dexamethasone, and thalidomide (17%). 3) Bortezomib, cyclophosphamide, and thalidomide (9%). 4) Dexamethasone and thalidomide (3%). 5) ASCT (1%).
Qasem, 2022	Jordan	Patients with newly diagnosed MM between January 2009 and the end of 2016	NR	Mean 36.8 months with SD 34.4 months. Median follow-up duration is 32 months	treatment with different regimens	# Two different first-line reimbursable regimens: 1) Dexamethasone and thalidomide (DT). 2) Bortezomib (Velcade), dexamethasone, and thalidomide (VDT). # Maintenance therapy with bortezomib post- ASCT for one year.
Mjali, 2021	Iraq	MM patients diagnosed between February 2012 to February 2020 at Al-Hussein cancer center	Patients with inconclusive results	Mean duration of follow-up was 24.67 months	Various treatment protocols	Various treatment protocols including VRD, VD, VCD, VTP, melphalan & prednisolone, supportive treatment, and VAD
Vasquez, 2021	Peru	Patients with a diagnosis of MM at Instituto Nacional de Enfermedades Neoplásicas, having received at least four cycles of CTd (cyclophosphamide, thalidomide, dexamethasone)	Previously treated MM, other cancers, HIV infection, plasma cell leukemia, incomplete medical records	Median follow-up was 41 months (range 5-138 months)	CTd regimen	cyclophosphamide 400 mg/m² orally for 5 days, thalidomide 100 mg increasing to 200 mg if tolerated, dexamethasone 40 mg weekly, 28-day cycles
Leon-Rodriguez, 2020	Mexico	Patients with MM undergoing auto-HSCT at the National Institute of Medical Sciences and Nutrition “Salvador Zubiran” in Mexico City, between March 2007 to March 2018	Over 65 years of age, minimal response, stable, active or progressive disease prior to auto-HSCT	Not explicitly stated, but survival data are provided up to 5 years	Auto-HSCT	Auto-HSCT with peripheral blood stem cells, Melphalan 200 mg/m² as conditioning regimen
Nair, 2019	India	Newly diagnosed MM patients who were transplant-eligible, treated between January 2011 to June 2017	Over 65 years, minimal response, stable, active or progressive disease before auto-HSCT, other cancers, HIV infection, diagnosis of plasma cell leukemia, incomplete medical records	Median follow-up 51 months	auto-HSCT	Induction chemotherapy for 4–6 cycles followed by ASCT with high dose Melphalan as conditioning chemotherapy
Bourlon, 2015	Mexico	Patients diagnosed with MM at a clinic in Mexico City between June 2006 and December 2014 who were prospectively followed	Patients that did not receive therapy, were not evaluated, or had less than 1 month of follow-up were excluded from the survival analysis	Median follow-up was 20 months (range, 2-104 months)	Various induction regimens	Various induction regimens were used: 21% melphalan-based, 68% thalidomide-based, and 11% Bortezomib-based. Only 6% of the patients received ASCT
Nair, 2015	India	All newly diagnosed MM cases from January 2011 to December 2014 who did not undergo stem cell transplant	Not explicitly stated, but implied that those who underwent a stem cell transplant were excluded	Median follow-up duration was 22 months	Novel agents (lenalidomide, thalidomide, and bortezomib)	Treatment with generic forms of novel agents (lenalidomide, thalidomide, and bortezomib) for transplant-ineligible patients
Wadhwa, 2002	India	Newly diagnosed and previously untreated patients with MM who received sequential chemotherapy with VAD followed by VMCP	Not explicitly stated, but possibly includes patients who did not receive the sequential chemotherapy regimen as per the study protocol	NR	VAD followed by VMCP	VAD (Vincristine, Doxorubicin, Dexamethasone) followed by VMCP (Vincristine, Melphalan, Cyclophosphamide, Prednisolone) as per a specified schedule
Martinez, 2022	Brazil	All patients with newly diagnosed MM treated at Hospital das Clinicas de São Paulo Complex between 2009 and 2019	NR	Median follow-up time was 5 years	Different regimens	Main frontline regimen was cyclophosphamide, dexamethasone, and thalidomide. Alkylating agent + dexamethasone regimens were also used. Only three patients received bortezomib upfront. 379 patients were referred for ASCT, and 257 proceeded with it
Hewamana, 2022	Sri Lanka	Patients with a diagnosis of plasma cell myeloma (PCM) who presented to LHBCC and completed the first-line therapy between May 1, 2013, and September 30, 2021	NR	Median follow-up of 40.63 months (95% CI, 35.2 to 59.07)	CTD or BTD	Treatments included 28-day cycles of CTD (cyclophosphamide, thalidomide, and dexamethasone) or BTD (bortezomib, thalidomide, dexamethasone)
Salgado, 2021	Brazil	Symptomatic MM patients receiving up-front CTD or VCD and undergoing ASCT	NR	Median treatment time with Lenalidomide maintenance was mentioned to be 20.5 months for a subset of patients	Various treatment protocols	Patients received up-front CTD (cyclophosphamide, thalidomide, dexamethasone) or VCD (bortezomib, cyclophosphamide, dexamethasone), followed by ASCT and MRD-NGF monitoring at day 100 post-transplant. A subset received maintenance therapy with Lenalidomide
Vasquez, 2021	Peru	Patients with relapsed/refractory MM eligible for ASCT, treated at Instituto Nacional de Enfermedades Neoplásicas between January 2014 and December 2016	NR	Median follow-up of 19 months for VTd regimen and 41 months since MM diagnosis	Bortezomib-Thalidomide-Dexamethasone (VTD) regimen	Bortezomib: 1.3 mg/m² subcutaneously on days 1, 4, 8, and 11. Thalidomide: 100 mg orally on days 1-21. Dexamethasone: 40 mg orally on days 1, 2, 4, 5, 8, 9, 11, and 12. Cycles: Three-week cycles. Additional prophylaxis: Antithrombotic (acetylsalicylic acid), herpes zoster (acyclovir), and trimethoprim/sulfamethoxazole.
Vargas-Serafin, 2021	Mexico	Adults (≥18 years old) with newly diagnosed between January 2006 and December 2018	Patients with incomplete records	Median 22.5 months (range 0.5–150 months)	Induction regimens including alkylating agents, thalidomide, or bortezomib-based therapies	Alkylating agents: 18.2%. Thalidomide-based regimens: 67.8%. Bortezomib-based regimens: 13.1%.
Pydi, 2019	India	Patients diagnosed with MM from 2013 to 2018, who progressed on first-line therapy or had responses of less than partial remission (PR).	NR	Median follow-up: 18 months	Second-line and later lines of therapy	Second-line therapy initiated for patients progressing on first-line therapy. Regimens included triplet chemotherapy, with MPT (melphalan, prednisone, thalidomide) and VRD (bortezomib, lenalidomide, dexamethasone) being the most commonly used. ASCT was performed in 17.2% of patients receiving second-line therapy.
Kumar, 2019	India	Patients with MM undergoing ASCT as part of remission consolidation therapy	NR	Median follow-up of 45.3 months (range: 12.1-74.2 months)	Bortezomib-based three-drug induction regimen followed by ASCT	Induction therapy: Bortezomib, Thalidomide or Lenalidomide, and Dexamethasone. High-dose Melphalan for conditioning. Maintenance therapy post-ASCT with Thalidomide, Lenalidomide, or Bortezomib.
Riva, 2019	Uruguay	Adults with symptomatic MM diagnosed and treated between January 2012 and January 2015	Patients with smoldering MM or institutions that did not consent to data review	Median follow-up of 32 months	Frontline treatment options (e.g., thalidomide, bortezomib-based therapies, ASCT)	Varied frontline treatments based on local guidelines, including bortezomib for high-risk MM, thalidomide, and conventional chemotherapy regimens
Sossa, 2019	Colombia	Patients with a diagnosis of MM documented in the RENEHOC registry during 2018	NR	Median overall survival (OS) reported as 56.5 months	Various induction therapies, including CyBorD, VTD, Bortezomib-Dexa, and VISTA; consolidation with ASCT	Chemotherapy regimens and ASCT for eligible patients
Kulkarni, 2019	India	Patients with MM undergoing ASCT from January 1995 to December 2014	NR	Median follow-up was 40.7 months (range 0–237.4 months)	ASCT	ASCT involved peripheral blood stem cell mobilization with GCSF, melphalan as a conditioning agent, and non-cryopreserved graft infusion for most patients
Kulkarni, 2018	India	Patients with newly diagnosed MM undergoing ASCT using granulocyte colony-stimulating factor (G-CSF) mobilized non-cryopreserved grafts.	Use of cyclophosphamide mobilization; Complications after stem cell harvest requiring cryopreservation	Survival assessed at 5 years	G-CSF mobilized non-cryopreserved grafts	G-CSF (5 μg/kg twice daily for 4 days) followed by apheresis. Peripheral blood stem cells (PBSCs) stored at 4°C for up to 72 hours. Transplantation conducted after high-dose melphalan chemotherapy.
Sossa, 2018	Colombia	Patients who underwent ASCT between November 2009 and December 2017	NR	Outcomes measured at 100 days, one year, and five years	ASCT	Graft Source: Peripheral blood stem cells for all patients. Conditioning Regimens: High-dose melphalan (MEL 200) for MM. BEAM regimen (carmustine, etoposide, cytarabine, melphalan) for lymphoma.
Hameed, 2018	Pakistan	Histopathological diagnosis of MM. Receipt of chemotherapy in the hospital.	Patients lost to follow-up before treatment completion. Patients with isolated plasmacytoma or asymptomatic myeloma (smoldering myeloma).	Overall survival (OS) median: 48 months	CTD (cyclophosphamide, thalidomide, dexamethasone). VTD (bortezomib, thalidomide, dexamethasone). Other regimens including thalidomide with dexamethasone or melphalan-based regimens.	Combination chemotherapy regimens with a focus on cost-effectiveness (CTD) and limited use of bortezomib-based regimens due to financial constraints
Jalaeikhoo, 2018	Iran	Newly diagnosed MM cases with at least 10% plasma cells in bone marrow biopsy and one CRAB finding (hypercalcemia, renal failure, anemia, or myeloma bone lesions)	NR	Median follow-up: 45 months (approximately 195 weeks)	Various chemotherapy regimens, including vincristine-adriamycin-dexamethasone (VAD), mini-CHOP, and bortezomib-based therapies. Some patients received ASCT	VAD, mini-CHOP, and bortezomib were used as first-line treatments. Maintenance therapy included thalidomide or lenalidomide. For skeletal care, pamidronate and zoledronic acid were used
Jacob, 2017	India	Patients diagnosed with symptomatic MM from 2005 to 2016 based on International Myeloma Working Group criteria	NR	Median duration of treatment was 9 months for the TD regimen and 7 months for the VTD regimen. Median survival ranged from 21 to 55 months depending on ISS stage and treatment regimen	Thalidomide + Dexamethasone (TD) and Bortezomib + TD (VTD)	TD regimen was used in 58% of patients. VTD regimen was used in 24% of patients. Proteasome inhibitors improved response and survival rates.
Salgado, 2023	Brazil	Patients with MM who underwent ASCT and Day +100 post-ASCT MRD assessment	Cases without sufficient sample for MRD were excluded	Median follow-up of 34 months	Lenalidomide Maintenance (M-Len)	Continuous lenalidomide maintenance therapy post-ASCT, with MRD monitoring using next-generation flow cytometry
Omoti, 2007	Nigeria	Diagnosis of MM based on atypical plasmacytosis (≥30% in bone marrow), presence of monoclonal proteins in serum or urine, and/or skeletal lytic lesions	NR	Up to 10 years (range: 1–96 months)	VAD (Vincristine, Adriamycin, Dexamethasone) MP (Melphalan, Prednisolone) Combined MP and VAD No therapy (NONE).	Patients were treated with either MP or VAD regimens, or a combination of both. Those receiving combination therapy showed improved survival compared to single regimen or no treatment.
Tarín-Arzaga, 2018	Mexico	Patients newly diagnosed with symptomatic MM from October 2007 to July 2016	Patients lost to follow-up before first disease evaluation (n=8). Patients treated at another institution (n=10).	Median follow-up: 41 months (range: 31-51 months)	Comparison of treatment regimens between public (PubC) and private (PrivC) healthcare systems	Public cohort (PubC): Primarily treated with a thalidomide-based regimen (cyclophosphamide, thalidomide, dexamethasone). Private cohort (PrivC): Primarily treated with bortezomib-based regimens (e.g., bortezomib, cyclophosphamide, dexamethasone).
Kumar, 2011	India	Patients undergoing ASCT between April 1990 and December 2009. Diagnoses included MM, lymphoma (Hodgkin’s and non-Hodgkin’s), leukemia, and certain solid tumors. Chemo-sensitive disease defined as CR, PR, or VGPR.	Patients with chemo-refractory disease unless indicated otherwise	Median follow-up of 66 months (range: 9–234 months)	ASCT	High-dose chemotherapy followed by infusion of autologous stem cells. Conditioning regimens varied by diagnosis and included agents such as melphalan, Bu-Cy2, BEAM, and others
Kumar, 2009	India	Patients with MM undergoing ASCT	NR	Median follow-up was 70 months (range, 9–142 months)	High-dose chemotherapy with ASCT	High-dose melphalan (200 mg/m²) followed by reinfusion of autologous peripheral blood stem cells
Bekadja, 2016	Algeria	Patients with MM eligible for ASCT (younger than 65 years, induction therapy completed, and high-dose chemotherapy)	NR	Median follow-up of 35 months (range: 3–75 months)	Non-cryopreserved ASCT	Stem cells mobilized using G-CSF (10 μg/kg/day for 5 days). Leukapheresis performed on day -2 and -1. Grafts stored at +4°C and reinfused on day 0. Conditioning regimen: melphalan 200 mg/m².
Bekadja, 2015	Algeria	Patients undergoing ASCT for MM	NR	Median of 35 months (range: 3 to 75 months)	Non-cryopreserved ASCT	Stem cells mobilized with G-CSF alone (10 μg/kg/day for 5 days). Leukapheresis performed on days -2 and -1. Grafts stored at +4°C in a blood bank refrigerator. Conditioning regimen: Melphalan 200 mg/m². Reinfusion on day 0.
Ali, 2015	Pakistan	Patients with MM under 65 years of age. Evidence of chemosensitive disease prior to transplant.	NR	Median: 66.3 months (range: 43.8-88.7 months)	ASCT	Noncryopreserved and unmanipulated peripheral blood stem cells mobilized using Filgrastim. High-dose Melphalan (100 mg/m²/day) as the conditioning regimen.
Nwabuko, 2014	Nigeria	Diagnosed and managed MM patients between 2003 and 2013	NR	Median survival interval of 39.2 months	Melphalan plus Prednisolone (MP) and Cyclophosphamide plus Prednisolone (CP), with additional VMP regimens	Chemotherapeutic regimens included MP, CP, and MP plus Bortezomib (V) triple combination
Kumar, 2013	India	Newly diagnosed symptomatic MM patients	NR	5 years (for overall survival and progression-free survival)	VD regimen (Bortezomib + Dexamethasone) followed by ASCT	Induction therapy: Bortezomib (1.3 mg/m²) and dexamethasone (40 mg) weekly for 4 cycles Stem cell mobilization: G-CSF (300 µg twice daily for 5 days) Conditioning regimen: High-dose melphalan (200 mg/m²) Post-transplant maintenance: Thalidomide or lenalidomide
Bekadja, 2012	Algeria	Age <65 years. Symptomatic MM Stage II or III. No major comorbidities. Normal renal and liver function tests.	Major comorbidities. Abnormal renal or liver function tests.	Median: 10 months (range: 3–30 months)	High-dose chemotherapy and ASCT using non-cryopreserved autologous peripheral blood stem cells (PBSCs)	PBSCs mobilized using granulocyte colony-stimulating factor (G-CSF) and stored at 4°C in a blood bank refrigerator for up to 4 days. Conditioning regimen: Melphalan (200 mg/m²). No post-transplant G-CSF support. Prophylactic antibiotics, antivirals, and antifungals used during post-transplant recovery.

ASCT: Autologous stem cell transplantation; MM: Multiple myeloma

**Table:** Patient Characteristics:

Author, year	Number of participants	Age	Female%	Multiple Myeloma Stage	Genetic Abnormalities	Type of MM	Comorbidities
Hewamana, 2022	20	median 57 range 41 - 66 years	47%	NR	NR	NR	NR
Reddy, 2022	46	Median age of 51 years (range 45–68 years)	32.20%	NR	Thirteen (28.2%) patients had high-risk cytogenetics	NR	NR
Sasmith, 2022	70	Median age of 55 years (range 38-88)	41.70%	The Revised International Staging Syndrome (ISS) at diagnosis: ISS 1 (34%), ISS 2 (31%), ISS 3 (35%)	NR	Most common type of MM was IgG myeloma (77%)	NR
Qasem, 2022	128	63.3 (mean)	50%	ISS Stage I (27.4%), Stage II (36.28%), and Stage III (36.28%)	NR	79.1% IgG type, 13.9% IgA type, and 7% light chain type	NR
Mjali, 2021	78	Median age: 59.8 years (range 33-91 years)	53.85%	NR	NR	Paraprotein types: IgG (61.53%), IgA (19.24%). Immunofixation: kappa chain (57.70%), lambda chain (37.17%), non-secretary (5.13%).	NR
Vasquez, 2021	59	Median age: 56 years (range 27-78)	41%	Predominance of early stages, based on the International Staging System	NR	NR	NR
Leon-Rodriguez, 2020	42	Median age: 53 years (range 37-64)	43%	ISS at diagnosis: I (26%), II (31%), III (38%)	NR	Subtype: IgG kappa 45%, IgG lambda 21%, IgA kappa 9%, IgA lambda 16%, Light chains 9%	Obesity 21%, DM2 17%, Dyslipidemia 26%
Nair, 2019	89 patients were eligible; 23 underwent auto-HSCT	Median age of the entire group: 57 years (range 32–66)	55%	ISS Stage I (26%), Stage II (31%), and Stage III (43%)	NR	NR	34% had some form of comorbidities, with diabetes being the most common (19%)
Bourlon, 2015	175 patients diagnosed, 152 received treatment, and 132 were included in the survival analysis	Median age: 62 years (range, 35-92 years)	56%	Most patients were at an advanced stage, with 88% in ISS categories II and III	NR	NR	NR
Nair, 2015	119	62 years (range 44-85)	55.50%	18.4% stage 1, 30% stage 2, 32.7% stage 3; missing data in 22 patients	NR	IgG (70%), IgA (17.6%), light chain (11.7%)	Comorbidities were present in 47.9% of patients
Wadhwa, 2002	28	Median age at diagnosis: 55 years (range: 29–70 years)	25%	NR	NR	NR	NR
Martinez, 2022	816	Mean age: 63 years	45%	Clinical stages at diagnosis: 88% Durie Salmon III, 46% ISS III	NR	NR	NR
Hewamana, 2022	79	Median age: 64 years	47%	NR	NR	NR	Hypercalcemia (36.7%), renal impairment (38%), anemia (72.1%), and bone disease (81%)
Salgado, 2021	53	Median age: 58 years (range 40-70)	51%	NR	NR	NR	NR
Vasquez, 2021	16	Median: 52 years (range: 39–62 years)	44% (7 females)	75% of participants had International Staging System (ISS) III disease	NR	IgG (62.5%) and IgA (12.5%)	NR
Vargas-Serafin, 2021	245	Median 62 years (range 35–92 years)	51%	ISS 1: 10.6%; ISS 2: 31%; ISS 3: 58.4%	High-risk cytogenetics in 31.6%	IgG: 51.8%; IgA: 21.6%; Free light chain (FLC): 22.5%; Other: 4.1%	Charlson comorbidity index (CCI) ≥2 in 64.1%; Comorbidities in 69.4%
Pydi, 2019	258 [Patients receiving second-line therapy: 81 (31% of total)]	Median age at presentation: 56 years (range: 28–84 years)	33%	NR	NR	NR	NR
Kumar, 2019	66	Median: 57 years (Range: 22-66 years)	36% (24 females)	ISS Stage III: 58% of patients	NR	IgG Kappa: 47%	Anemia: 59%; Bone disease: 70%; Renal failure: 35%
Riva, 2019	222	Median 66 years (range 33–94)	45.50%	79.5% diagnosed at Durie-Salmon Stage III, and 48% at ISS Stage 3	High-risk features detected in 6% by conventional cytogenetics and 18.2% by FISH	IgG (50.9%), IgA (23%), light chains (18.5%), non-secretor (2.3%), IgM (<1%)	NR
Sossa, 2019	334	Mean age at diagnosis: 64.4 years (SD ± 11.09)	44.10%	Majority were advanced by Durie-Salmon staging (IIIA or IIIB: 64%)	Molecular cytogenetic prognostic characterization was available for 20% of patients	IgG monoclonal component most common (52.7%)	Renal failure in 25.1%; 34% of these required dialysis
Kulkarni, 2019	245	Median: 51 years (range: 23–68)	31%	NR	NR	NR	NR
Kulkarni, 2018	224	Median: 50 years (range: 23–68)	30.40%	International Staging System: Stage 1: 31.4%; Stage 2: 33.9%; Stage 3: 34.7%	NR	NR	NR
Sossa, 2018	157	Mean: 49.54 years (range 14–71)	49.69%	NR	NR	NR	NR
Hameed, 2018	82	Median: 51 years (Range: 23-64 years)	43.90%	Stage I: 25.7%; Stage II: 34.1%; Stage III: 40.2%	NR	IgG: 58.5%; IgA: 18.3%; Light Chain: 14.6%; Non-Secretory: 8.5%	NR
Jalaeikhoo, 2018	345	Mean age: 61.98 ± 11.44 years (range: 30–88 years)	36.50%	NR	NR	NR	NR
Jacob, 2017	389	Median age: 54 years (range: 39–85 years)	32%	ISS I: 31%; ISS II: 30%; ISS III: 39%	NR	IgG: 72%; IgA: 10%; IgM: 2%; Light chain disease: 16%	NR
Salgado, 2023	53	Median 58 years (range 40–70 years)	51%	Durie-Salmon (DS) Stage III: 68%. International Staging System (ISS): Stage I (40%), Stage II (32%), Stage III (28%).	NR	IgG (63%), IgA (15%), light chain (17%), non-secretory (3%)	NR
Omoti, 2007	30	Median 54 years (range 34–75)	33.30%	NR	NR	NR	NR
Tarín-Arzaga, 2018	148 patients (PubC: 77, PrivC: 71)	Median: 60 years (IQR: 54-71 years). PubC: Median 59 years (IQR: 51-74 years). PrivC: Median 61 years (IQR: 54-70 years).	44%	ISS Stage I: 26% (PubC: 21%, PrivC: 34%). ISS Stage II: 34% (PubC: 28%, PrivC: 38%). ISS Stage III: 40% (PubC: 51%, PrivC: 28%).	NR	IgG: 58% IgA: 25% Light chain: 15% IgM and nonsecretory: <1% each.	NR
Kumar, 2011	228	Median 48 years (range: 11–68)	30.70%	NR	NR	NR	NR
Kumar, 2009	108	Median 52 years (range, 26–68 years)	27.80%	Stage IIIA: 73.1%; Stage IIIB: 22.2%	NR	Subtypes include IgG kappa (45.4%), IgG lambda (20.4%), IgA kappa (7.4%), IgA lambda (7.4%), kappa light chain (6.5%), lambda light chain (5.6%), non-secretory (1.9%)	Renal insufficiency at diagnosis in 22.2% of patients
Bekadja, 2016	134	Median age: 55 years (range: 27–67 years)	40.30%	NR	NR	NR	NR
Bekadja, 2015	134	Median: 55 years (range: 27–67 years)	40.30%	NR	NR	NR	NR
Ali, 2015	10	Median 48 years ± 7.5 (range: 32-55 years)	30%	NR	NR	NR	NR
Nwabuko, 2014	26	Median 60.6 years	30.30%	61.5% Durie Salmon stage III, 30.8% stage II, 7.7% stage I	NR	NR	NR
Kumar, 2013	10	NR	NR	NR	NR	NR	NR
Bekadja, 2012	54	Median: 55 years (range: 35–65 years)	31.50%	Stage II: 11 patients; Stage III: 43 patients	NR	IgG: 29 cases; IgA: 16 cases; Light chain disease: 9 cases	NR

**Table 2.** Risk of Bias

Author, year	Representativeness of the cohort	Ascertainment of exposure	It is clear that the binary outcome of interest was not present at start of study	Ascertainment of outcome: Independent blind assessment	Follow-up long enough for outcomes to occur	Adequacy of follow-up	Is the study described with sufficient details to allow other investigators to replicate the research or to allow practitioners make inferences related to their own practice?
Hewamana, 2022	Low risk	Low risk	Yes	Low risk	Low risk	Low risk	High risk
Reddy, 2022	Low risk	Low risk	Yes	Low risk	High risk	Low risk	High risk
Sasmith, 2022	Low risk	Low risk	Yes	Unclear	Low risk	Unclear	High risk
Qasem, 2022	Low risk	Low risk	Yes	Low risk	Low risk	Low risk	Low risk
Mjali, 2021	Low risk	Low risk	Yes	Low risk	Low risk	Low risk	Low risk
Vasquez, 2021	Low risk	Low risk	Yes	Low risk	Low risk	Unclear	Low risk
Leon-Rodriguez, 2020	Low risk	Low risk	Yes	Low risk	Low risk	Low risk	High risk
Nair, 2019	Low risk	Low risk	Yes	Low risk	Low risk	Low risk	Low risk
Bourlon, 2015	Low risk	Low risk	Yes	Low risk	High risk	Low risk	High risk
Nair, 2015	Low risk	Low risk	Yes	Low risk	High risk	Low risk	Low risk
Wadhwa, 2002	Low risk	Low risk	Yes	Low risk	Unclear	Unclear	Low risk
Martinez, 2022	Low risk	Low risk	Yes	Low risk	Low risk	Unclear	High risk
Hewamana, 2022	Low risk	Low risk	Yes	Low risk	Low risk	Unclear	Low risk
Salgado, 2021	Low risk	Low risk	Yes	Unclear	High risk	Unclear	High risk
Vasquez, 2021	Low risk	Low risk	Yes	Unclear	Low risk	Unclear	Low risk
Vargas-Serafin, 2021	Low risk	Low risk	Yes	Unclear	Low risk	Unclear	Low risk
Pydi, 2019	High risk	Low risk	Yes	Unclear	Low risk	Unclear	Low risk
Kumar, 2019	High risk	Low risk	Yes	Unclear	Low risk	Unclear	Low risk
Riva, 2019	High risk	Low risk	Yes	Unclear	Low risk	High risk	Low risk
Sossa, 2019	Low risk	Low risk	Yes	Unclear	Low risk	Low risk	Low risk
Kulkarni, 2019	Low risk	Low risk	Yes	Low risk	Low risk	Low risk	Low risk
Kulkarni, 2018	Low risk	Low risk	Yes	Low risk	Low risk	Low risk	Low risk
Sossa, 2018	Low risk	Low risk	Yes	Low risk	Low risk	Low risk	Low risk
Hameed, 2018	Low risk	Low risk	Yes	Unclear	Low risk	Low risk	Low risk
Jalaeikhoo, 2018	Low risk	Low risk	Yes	Unclear	Low risk	Low risk	Low risk
Jacob, 2017	Low risk	Low risk	Yes	Unclear	Low risk	Low risk	Low risk
Salgado, 2023	Low risk	Low risk	Unclear	Low risk	Low risk	Low risk	Low risk
Omoti, 2007	Low risk	Low risk	Yes	Low risk	Low risk	Unclear	Low risk
Tarín-Arzaga, 2018	Low risk	Low risk	Yes	Low risk	Low risk	Low risk	Low risk
Kumar, 2011	Low risk	Low risk	Yes	Low risk	Low risk	Low risk	Low risk
Kumar, 2009	Low risk	Low risk	Yes	Low risk	Low risk	Low risk	Low risk
Bekadja, 2016	Low risk	Low risk	Yes	Unclear	Low risk	Low risk	Low risk
Bekadja, 2015	Low risk	Low risk	Yes	Unclear	Low risk	Low risk	Low risk
Ali, 2015	Low risk	Low risk	Yes	Unclear	Low risk	Low risk	Low risk
Nwabuko, 2014	Low risk	Low risk	Yes	Unclear	Low risk	Unclear	Moderate risk
Kumar, 2013	Low risk	Low risk	Yes	Unclear	Low risk	Low risk	Low risk
Bekadja, 2012	Low risk	Low risk	Yes	Low risk	High risk	Low risk	Low risk

Treatment approaches included conventional chemotherapy, immunomodulatory drugs (thalidomide, lenalidomide), proteasome inhibitors (bortezomib), and ASCT. Notably, anti-CD38 monoclonal antibodies (daratumumab, isatuximab) and newer proteasome inhibitors (carfilzomib) were absent. Pomalidomide-based regimen was used as a salvage regimen post ASCT relapse in one study. Follow-up durations ranged from 8 months to 12.5 years, with most studies (n=17) reporting median follow-ups of 2-5 years.

Regional subgroup analysis revealed notable variations in outcomes. Asian countries (primarily India and Sri Lanka, n=13 studies) showed pooled OS rates at longest follow-up of 58% (95%CI: 41-74%) for ASCT patients, while Latin American countries (Brazil, Mexico, Colombia, n=11studies) demonstrated higher rates of 68% (95% CI: 52-81%). African studies (Algeria, n=3studies) showed intermediate outcomes with pooled OS of 62% (95% CI: 45-77%). These differences likely reflect variations in healthcare infrastructure, treatment accessibility, and patient selection criteria across regions.

### Overall Survival with ASCT

**Figure 2.**
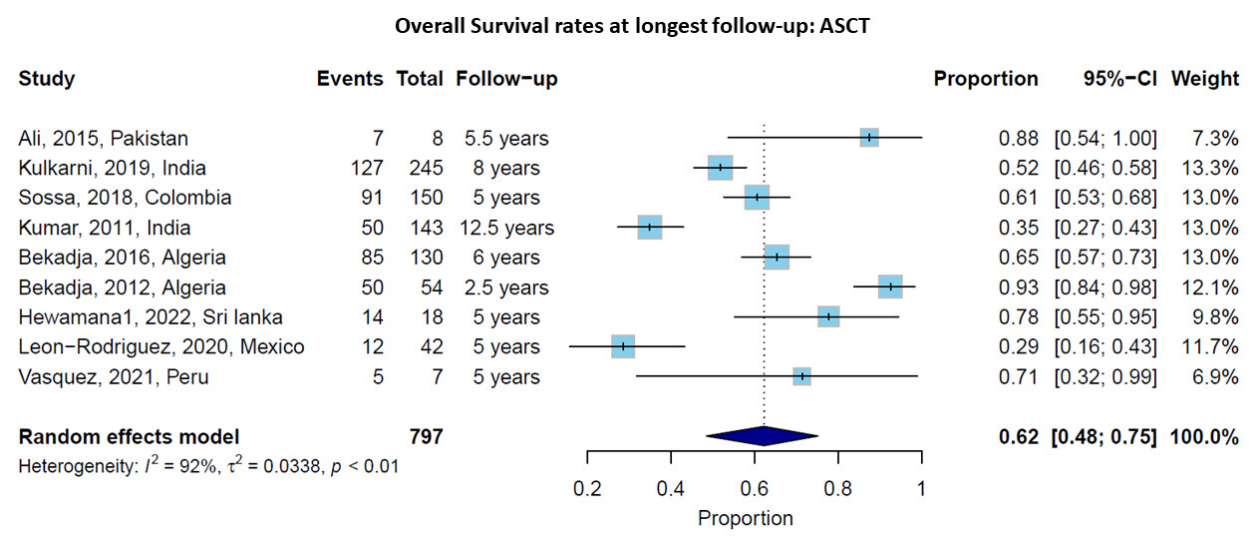
Overall Survival rates at longest follow-up for patients receiving autologous stem cell transplantation (ASCT). Forest plot showing individual studies and the pooled estimate with 95% confidence interval.

Nine studies provided data on OS rates at longest follow-up (ranging from 2.5 to 12.5 years) for patients undergoing ASCT. The pooled OS rate at the longest follow-up (ranging from 2.5 to 12.5 years) was 62% (95% CI: 48-75%), with significant heterogeneity across studies (I² = 92%, p < 0.01) (**Figure 2**). Survival rates varied considerably by country and follow-up duration, ranging from 29%^4^ to 93%.^5^ Studies with longer follow-up generally reported lower survival rates, as expected. For instance, Kumar et al.^6^ reported a 12.5-year OS rate of 35%, while studies with follow-up of 5-6 years reported OS rates of 60-78%.

Sensitivity analysis excluding smaller studies (n<50 patients) showed similar pooled OS rates for ASCT (64% versus 62%), confirming the robustness of our findings. Studies published after 2015 showed slightly better outcomes than earlier studies (65% versus 58% OS), likely reflecting improved supportive care and treatment protocols over time.

High-quality studies (Newcastle-Ottawa Scale score ≥7, n=27) showed findings consistent with the overall analysis, with pooled OS rates of 63% for ASCT patients, supporting the robustness of our conclusions despite inclusion of some lower-quality studies.

The median OS for ASCT patients was reported in several studies, ranging from 56.5 months^7^ to 70.8 months,^8^ with some studies reporting that median OS was not reached during their follow-up period, suggesting favorable outcomes.

### Progression-Free Survival with ASCT

**Figure 3.**
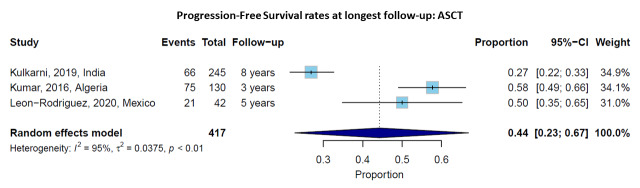
Progression-Free Survival rates at longest follow-up for patients receiving autologous stem cell transplantation (ASCT). Forest plot showing individual studies and the pooled estimate with 95% confidence interval.

Three studies reported PFS rates at longest follow-up for ASCT patients. The pooled PFS rate at the longest follow-up (ranging from 3 to 8 years) was 44% (95% CI: 23-67%), with high heterogeneity (I² = 95%, p < 0.01) (**Figure 3**). Individual study rates ranged from 27% at 8 years^9^ to 58% at 3 years.^10^ The median PFS for ASCT patients ranged from 19 months^11^ to 46 months.^7^

### Comparative Outcomes: ASCT versus No ASCT

**Figure 4.**
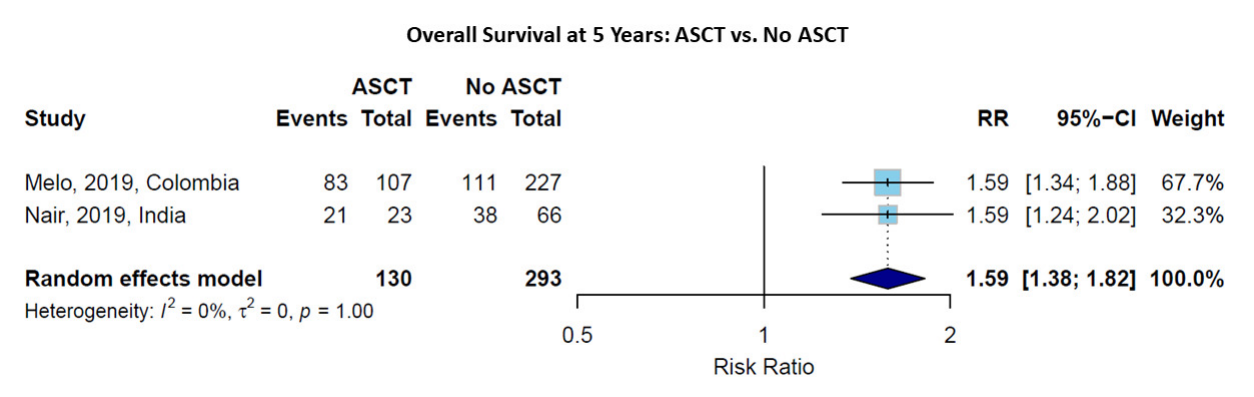
Risk ratios for Overall Survival at 5 years comparing ASCT versus non-ASCT treatment. Forest plot showing individual studies and the pooled estimate with 95% confidence interval.

Two studies^7,12^ provided comparative data on OS for patients receiving ASCT versus conventional chemotherapy with no ASCT. At 1 year, the pooled risk ratio for OS favoring ASCT was 1.11 (95% CI: 0.93-1.33), with substantial heterogeneity (I² = 96%, p < 0.01) (**Supplementary Figure S1**). However, the survival advantage of ASCT became more pronounced with longer follow-up. At 5 years, the pooled RR was 1.59 (95% CI: 1.38-1.82), with no heterogeneity (I² = 0%, p = 1.00) (**Figure 4**), indicating a consistent and significant association with better survival with the use of ASCT over conventional treatment.

For PFS, ASCT showed a significant advantage even at earlier time points. The RR at 1 year was 1.27 (95% CI: 1.10-1.47), increasing to 3.03 (95% CI: 1.96-4.69) at 5 years, based on data from Nair, et al (2019).^12^ Hazard ratios further confirmed this benefit, with HR for PFS of 0.09 (95% CI: 0.02-0.40, p = 0.001).

### Novel Agents and Other Treatment Approaches

Several studies evaluated outcomes with novel agents. Vargas-Serafin et al (2021)^13^ compared three regimens: bortezomib-based, thalidomide-based, and alkylating agent-based therapies. At 4 years, bortezomib-based therapy was associated with superior OS compared to alkylating agents (RR: 0.48; 95% CI: 0.28-0.83) and a trend toward superiority over thalidomide (RR: 0.73; 95% CI: 0.53-1.01).

Similarly, Jacob et al (2017)^14^ reported that bortezomib-thalidomide-dexamethasone (VTD) was associated with improvement in 5-year OS compared to thalidomide-dexamethasone (TD) (RR: 2.73; 95% CI: 1.09-6.87). This advantage was observed across all ISS stages but was most pronounced in advanced disease.

For maintenance therapy, Salgado et al (2023)^15^ showed that lenalidomide maintenance after ASCT improved both PFS (RR at 4 years: 2.43; 95% CI: 1.47-4.02) and OS (RR at 4 years: 1.63; 95% CI: 1.23-2.15) compared to no maintenance.

Non-comparative studies of various regimens showed that conventional chemotherapies like melphalan-prednisolone (MP) and vincristine-adriamycin-dexamethasone (VAD) achieved 1-year OS rates of 50-60%.^16^ In contrast, novel agent-based regimens achieved 1-year OS rates of 80-97%.^11,17^

### Subgroup Analyses

Limited subgroup data were available. Riva et al (2019)^18^ reported outcomes by age, with similar PFS at 3.3 years between patients <70 years (8%) and >70 years (13%). However, OS at 4 years was better in older patients (66%) compared to younger ones (37%), possibly reflecting selection bias for fitter elderly patients. Qasem et al (2022)^19^ reported outcomes by ISS stage, with median OS of 96 months for stage I, 46 months for stage II, and 16 months for stage III, highlighting the impact of disease stage on prognosis.

## DISCUSSION

This systematic review and meta-analysis provide the first quantitative synthesis of MM outcomes across developing countries, offering evidence-based benchmarks for healthcare planning and resource allocation. The pooled survival estimates and comparative effectiveness data can inform treatment guidelines and policy decisions specific to resource-limited settings. Our findings demonstrate that, despite resource constraints, significant improvements in survival were associated with ASCT. The pooled OS rate of 62% with ASCT at the longest follow-up is encouraging, though lower than those reported in developed countries, where 5-year OS rates following ASCT in patients treated after 2014 often exceed 70-80%.^20^

The regional variations observed in our analysis highlight the heterogeneity within LMICs. Latin American countries generally showed superior outcomes, possibly reflecting better healthcare infrastructure and earlier adoption of novel therapies compared to other regions. These findings underscore the need for region-specific strategies to improve MM care in developing countries.

The substantial benefit of ASCT over conventional chemotherapy with no ASCT, with a 59% increase in 5-year survival, aligns with findings from studies in high-income settings. This consistent benefit across different healthcare contexts underscores the importance of expanding transplant capabilities in developing regions. However, it is worth noting that only a small proportion of eligible patients in the included studies actually received ASCT, highlighting access barriers that persist in resource-limited settings.

The heterogeneity observed in our analysis warrants careful consideration. Survival outcomes varied dramatically across studies, likely reflecting differences in patient characteristics, healthcare infrastructure, treatment protocols, supportive care availability, and follow-up durations. Moreover, publication bias may have influenced our findings, as centers with better outcomes may be more likely to publish their results.

The observed heterogeneity, while substantial, provides important insights into the variability of MM outcomes across different resource settings. This variation emphasizes the need for context-specific treatment approaches and highlights opportunities for improvement in under performing regions.

Novel agents, particularly bortezomib-based regimens, demonstrated superior outcomes compared to conventional chemotherapy, consistent with global evidence. The trend toward improved survival with bortezomib over thalidomide suggests that incorporating proteasome inhibitors into treatment protocols could significantly benefit patients in developing countries. However, the higher cost of these agents remains a substantial barrier to widespread implementation.

The limited use of newer novel agents reflects multiple barriers in developing countries. Cost represents the primary obstacle, with daratumumab and carfilzomib costing United States dollars $100,000-150,000 annually compared to $10,000-20,000 for bortezomib-based regimens. Regulatory approval delays, with some agents approved 3-5 years later than in developed countries, further limit access. Supply chain challenges, including cold storage requirements for monoclonal antibodies and limited distribution networks, pose additional barriers. Healthcare provider training and infrastructure limitations for administering complex regimens also contribute to underutilization. Anti-BCMA therapies like CAR-T cells face even greater barriers due to manufacturing complexity and the need for specialized treatment centers.

Maintenance therapy with lenalidomide after ASCT showed promising results, doubling 4-year PFS rates. This finding is particularly relevant, because maintenance strategies are increasingly recognized as essential components of myeloma management but may be underutilized in resource-constrained settings due to cost and accessibility issues. The use of newer novel agents such as daratumumab, carfilzomib, and pomalidomide was notably limited across studies.

Supportive care practices varied considerably across studies and were often inadequately reported, representing a significant limitation. Where mentioned, infection prophylaxis protocols were inconsistent, with only 40% of studies reporting standardized antimicrobial prophylaxis during ASCT. Renal protection strategies were described in 25% of studies, primarily focusing on hydration and bisphosphonate use. The lack of standardized supportive care protocols likely contributed to outcome variability and represents an important area for improvement in developing countries.

Innovative implementation strategies could help bridge the treatment gap in developing countries. Telemedicine platforms have shown promise for MM monitoring and consultation with specialized centers, potentially reducing geographical barriers. Hub-and-spoke models, where tertiary centers support satellite facilities, could expand ASCT capabilities. Global pricing initiatives, including tiered pricing based on national income levels and generic drug development, could improve affordability. Regional procurement collaboratives could leverage collective bargaining power for better drug pricing. Training programs and twinning partnerships between developed and developing country centers could enhance local expertise and treatment capabilities.

Our study has several limitations. First, the included studies were predominantly retrospective cohorts with inherent selection biases. Second, reporting of outcomes was inconsistent across studies, limiting our ability to pool data for certain endpoints. Third, details on supportive care, management of complications, and adherence to treatment protocols were often lacking, yet these factors significantly impact outcomes. Finally, most studies came from upper-middle-income countries, with limited data from low-income settings, where challenges may be even more pronounced.

Despite these limitations, our findings have important implications for clinical practice and health policy in developing countries. They suggest that investments in transplant infrastructure and increased access to novel agents could substantially improve patient outcomes. Resource-stratified guidelines that account for varying levels of healthcare capacity could help optimize treatment selection based on local contexts.

Future research should focus on cost-effective approaches to MM management in resource-limited settings, innovative care delivery models that maximize available resources, and implementation strategies to overcome barriers to optimal care. Prospective registries with standardized outcome reporting would provide more robust data on real-world outcomes and help identify best practices applicable to developing regions.

## CONCLUSION

MM treatment outcomes in developing countries demonstrate the efficacy of ASCT and novel agents, though with considerable variability across settings. The substantial survival benefits associated with these approaches highlight the importance of expanding access to modern myeloma therapies in resource-constrained environments. Strategic investments in healthcare infrastructure, capacity building, and affordable treatment options are essential to bridge the gap in outcomes between developed and developing countries. Establishing prospective registries with standardized outcome reporting across developing countries would provide more robust real-world evidence and help identify best practices applicable to resource-limited settings. Such registries could facilitate international collaboration and guide evidence-based policy decisions for MM care in LMICs. By addressing these challenges systematically, the global disparity in multiple myeloma outcomes can potentially be reduced, bringing the benefits of therapeutic advances to patients regardless of geographic location or economic status.

### Previous presentation

Portions of this work were presented at the 2024 annual meeting of the American Society of Hematology December 6-9, 2025, in San Deigo, CA.

### Conflict of interest

**HM:** HM: Consultancy/Honoraria fees from BMS, Takeda, Janssen, Amgen, Sanofi, Forus, GSK, Pfizer, Research funding Janssen.

**GRM**: Honoraria for writing from MashupMD and Medscape. Institution has received funding from **Janssen** for his role as a site PI of a trial.

**SAH**: Consultancy/Honoraria fees from Janssen, Pfizer and Sanofi. Research funding: Janssen

### Authors’ Contribution - CRediT

Conceptualization: Jehad Almasri and Samer Al Hadidi;

Methodology: Jehad Almasri, Bashar Hasan, Lynn Warner and Samer Al Hadidi;

Formal analysis and investigation: Jehad Almasri, Bashar Hasan, Zin Tarakji, Faris W. Naffa’, Lynn Warner and Samer Al Hadidi;

Writing - original draft preparation: Jehad Almasri and Samer Al Hadidi;

Writing - review and editing: Jehad Almasri, Bashar Hasan, Zin Tarakji, Faris W. Naffa’, Lynn Warner, Hira Mian, Rajshekhar Chakraborty, Ghulam Rehman Mohyuddin, Zaid Abdel-Rahman, Samer Al Hadidi;

Funding acquisition: N/A;

Resources: Jehad Almasri, Bashar Hasan, Lynn Warner and Samer Al Hadidi;

Supervision: Hira Mian, Rajshekhar Chakraborty, Ghulam Rehman Mohyuddin, Zaid Abdel-Rahman, Samer Al Hadidi

## Supplementary Material

Supplementary Figure S1. Risk ratios for Overall Survival at 1 year comparing ASCT versus non-ASCT treatment. Forest plot showing individual studies and the pooled estimate with 95% confidence interval.
