# Supplementary figures and images for: Treatment Outcomes of Multiple Myeloma in Developing Countries: A Systematic Review and Meta-Analysis
Source: Clin Hematol Int. 2025 Oct 8;7(4):1–20. doi: 10.46989/001c.144582 (PMC12512897; doi:10.46989/001c.144582)

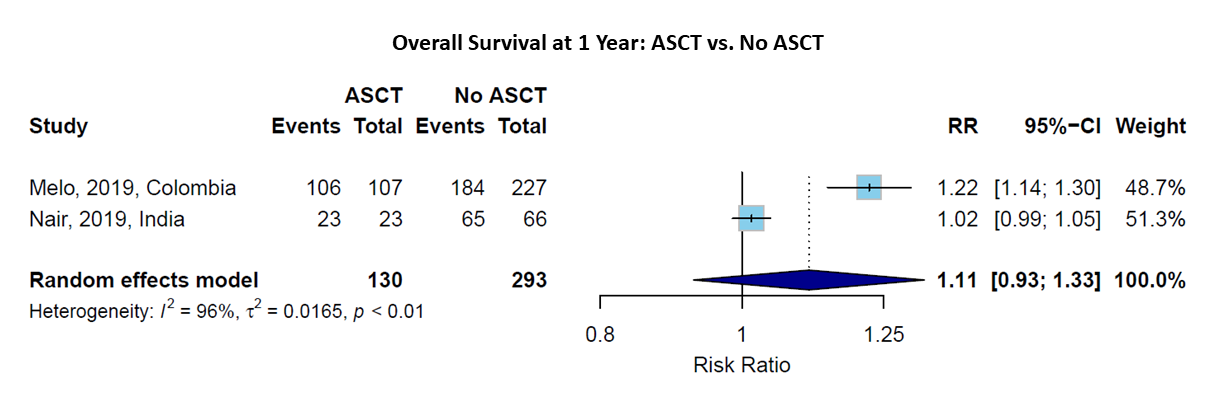

Supplement: Supplementary Figure S1. [file chi_2025_7_4_144582_302706.png]
